# Supplementary figures and images for: Use of hospital care services by chronic patients according to their characteristics and risk levels by adjusted morbidity groups
Source: PLoS One. 2022 Feb 3;17(2):e0262666. doi: 10.1371/journal.pone.0262666 (PMC8812854; doi:10.1371/journal.pone.0262666)

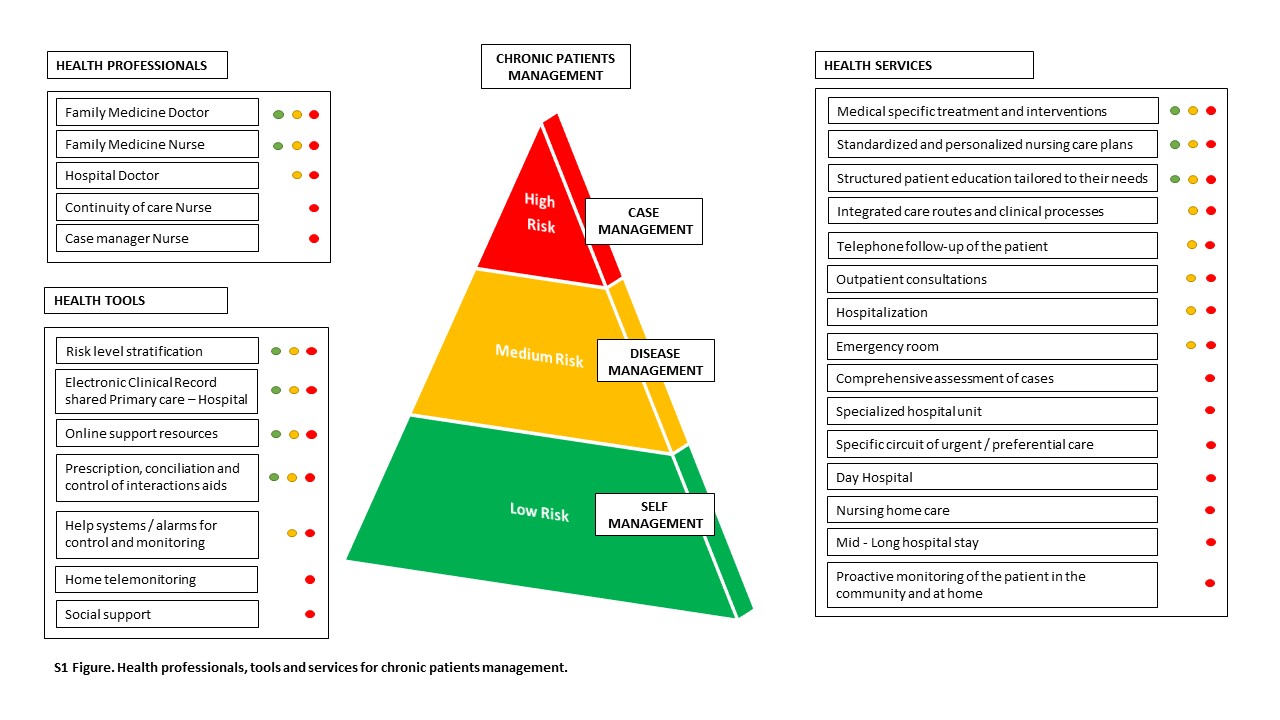

Supplement: S1 Fig — (JPG) [file pone.0262666.s001.jpg]
