# Supplementary material for: Use of hospital care services by chronic patients according to their characteristics and risk levels by adjusted morbidity groups
Source: PLoS One. 2022 Feb 3;17(2):e0262666. doi: 10.1371/journal.pone.0262666 (PMC8812854; doi:10.1371/journal.pone.0262666)
Supplement: S1 Table — (DOCX) [file pone.0262666.s002.docx]

**S1 Table. Types of chronic diseases considered by the Adjusted Morbidity Group (AMG) in the Community of Madrid at the time of data extraction**

| Alcoholism |
| --- |
| Anaemia |
| Aorta aneurysm |
| Anxiety |
| Arthritis |
| Arthrosis |
| Asthma |
| Attention-Deficit/Hyperactivity Disorder (ADHD) |
| Bladder cancer |
| Breast cancer |
| Cardiopulmonary disease |
| Central nervous system cancer |
| Cervical cancer |
| Cirrhosis |
| Colon cancer |
| Dementia |
| Depression |
| Diabetes Mellitus |
| Dyslipidaemia |
| Dysrhythmias |
| Ear, nose and throat cancer |
| Endometrial cancer |
| Epilepsy |
| Gastrointestinal ulcer |
| Glaucoma |
| Heart chronic failure |
| Hepatoblastoma |
| Hodgkin/Other lymphomas |
| Human immunodeficiency virus (HIV) |
| Hyperlipidemia |
| Hypertension |
| Ischemic heart disease |
| Leukemia |
| Liver cancer |
| Lung cancer |
| Mental retardation |
| Multiple sclerosis |
| Obesity |
| Obstructive chronic pulmonary disease (OCPD) |
| Osteoarthritis |
| Osteoporosis |
| Pancreatic cancer |
| Parkinson |
| Prostate cancer |
| Renal cancer |
| Renal chronic failure |
| Retinoblastoma |
| Schizophrenia |
| Skin cancer |
| Soft tissues cancer |
| Stomach cancer |
| Stroke |
| Substance abuse |
| Testicle cancer |
| Thyroid cancer |
| Thyroid disorder |
| Ulcerative colitis |
| Valvular heart disease |
| Vasculitis |
